# DNA damage response and repair genes in advanced bone and soft tissue sarcomas: An 8-gene signature as a candidate predictive biomarker of response to trabectedin and olaparib combination

**DOI:** 10.3389/fonc.2022.844250

**Published:** 2022-08-30

**Authors:** Alessandra Merlini, Maria Laura Centomo, Giulio Ferrero, Giulia Chiabotto, Umberto Miglio, Enrico Berrino, Giorgia Giordano, Silvia Brusco, Alberto Pisacane, Elena Maldi, Ivana Sarotto, Federica Capozzi, Cristina Lano, Claudio Isella, Giovanni Crisafulli, Massimo Aglietta, Angelo Paolo Dei Tos, Marta Sbaraglia, Dario Sangiolo, Lorenzo D’Ambrosio, Alberto Bardelli, Ymera Pignochino, Giovanni Grignani

**Affiliations:** ^1^ Candiolo Cancer Institute, FPO-IRCCS, Turin, Italy; ^2^ Department of Oncology, University of Torino, Turin, Italy; ^3^ Department of Clinical and Biological Sciences, University of Torino, Turin, Italy; ^4^ Department of Computer Science, University of Torino, Turin, Italy; ^5^ Department of Medical Sciences, University of Torino, Turin, Italy; ^6^ Department of Pathology, Azienda Ospedale-Università Padova, Padua, Italy; ^7^ Department of Medicine (DIMED), University of Padua School of Medicine, Padua, Italy; ^8^ Medical Oncology, AOU San Luigi Gonzaga, Orbassano (TO), Italy

**Keywords:** bone and soft tissue sarcomas, predictive biomarkers, DNA damage response and repair genes, trabectedin, olaparib

## Abstract

**Background:**

Advanced and unresectable bone and soft tissue sarcomas (BSTS) still represent an unmet medical need. We demonstrated that the alkylating agent trabectedin and the PARP1-inhibitor olaparib display antitumor activity in BSTS preclinical models. Moreover, in a phase Ib clinical trial (NCT02398058), feasibility, tolerability and encouraging results have been observed and the treatment combination is currently under study in a phase II trial (NCT03838744).

**Methods:**

Differential expression of genes involved in DNA Damage Response and Repair was evaluated by Nanostring^®^ technology, extracting RNA from pre-treatment tumor samples of 16 responder (≥6-month progression free survival) and 16 non-responder patients. Data validation was performed by quantitative real-time PCR, RNA *in situ* hybridization, and immunohistochemistry. The correlation between the identified candidate genes and both progression-free survival and overall survival was investigated in the publicly available dataset “Sarcoma (TCGA, The Cancer Genome Atlas)”.

**Results:**

Differential RNA expression analysis revealed an 8-gene signature (CDKN2A, PIK3R1, SLFN11, ATM, APEX2, BLM, XRCC2, MAD2L2) defining patients with better outcome upon trabectedin+olaparib treatment. In responder *vs.* non-responder patients, a significant differential expression of these genes was further confirmed by RNA *in situ* hybridization and by qRT-PCR and immunohistochemistry in selected experiments. Correlation between survival outcomes and genetic alterations in the identified genes was shown in the TCGA sarcoma dataset.

**Conclusions:**

This work identified an 8-gene expression signature to improve prediction of response to trabectedin+olaparib combination in BSTS. The predictive role of these potential biomarkers warrants further investigation.

## Introduction

Bone and soft tissue sarcomas (BSTS) are a wide and heterogeneous family of rare tumors sharing features of mesenchymal origin (1). In advanced stages, when the disease is unresectable or metastatic, prognosis is dismal. Medical treatment may delay progression, with marginal improvement in overall survival (OS) (2–5). This scenario is further complicated by the relative poorness of predictive factors to improve sarcoma patient selection for patient-tailored treatments, with the noteworthy exception of gastrointestinal stromal tumors (6). Hence, in the sarcoma field, there is a huge need to explore innovative therapies, focusing on combinations of cytotoxic compounds, target therapies and immunotherapeutic strategies, to optimize treatment personalization and increase tumor control in terms of mass shrinkage or - at least - sarcoma growth arrest. In recent years, several combinations have been tested (7–10) but, once again, these studies demonstrated promising results only in small subsets of patients. Hence, there is an urgent need to identify predictive biomarkers of tumor response to refine patient selection, according to the concept of precision medicine (11). In this perspective, we focused on the combination of trabectedin, an isoquinoline alkylating agent of marine origin, and the inhibitor of the enzyme poly-(ADP-ribose) polymerase 1 (PARP1) olaparib. Preclinical and clinical data confirmed feasibility and suggested hints of activity in a fraction of the enrolled patients, emphasizing the need to improve patient selection through the identification of specific predictive factors (12, 13). Both drugs under study – trabectedin and olaparib – had already been studied as single agents, looking for predictive factors among the tightly intertwined mechanisms ruled by DNA Damage Response and Repair (DDRR) genes (14–24). However, potential predictive factors of combined trabectedin+olaparib treatment response have not been investigated, so far.

Trabectedin creates DNA adducts by interfering with active transcription, wherein its activity is dependent on transcription-coupled nucleotide excision repair (TC-NER) (25–27). NER defects make tumor cells less sensitive to trabectedin damage and high expression levels of ERCC1 and XPG/ERCC5 (“signs” of a proficient NER machinery) have been described as predictive of better response to trabectedin treatment (14–16, 18). Tumor cells bearing homologous repair deficiency (HRD) are more sensitive to trabectedin-induced cell death, as they cannot recruit the proper machinery to repair the double-strand breaks (DSBs) generated upon trabectedin treatment. Non-homologous end joining (NHEJ) defects, instead, seem to have only a minor effect on trabectedin efficacy (14–16, 28). At present, none of these potential predictive factors of response upon trabectedin treatment has received approval for clinical use.

Differently, PARP1-inhibitors (PARP1i) have been marketed with specific indications with respect to homologous recombination (HR) status, which is considered a predictive biomarker of response to PARP1i (24, 29, 30). The European Medicine Agency has approved olaparib use in ovarian cancer with HRD, while for pancreatic, prostate and breast cancers the indication more strictly refers to *BRCA1/2-*mutated patients. Hence, *BRCA* mutational status and HRD are clinical-grade approved predictive biomarkers, to better select patients who might benefit more from PARP1i treatment. Indeed, in cells showing HRD, NHEJ takes action upon DSBs formation, but with respect to HR, considered “error-free”, NHEJ provides DSBs repair with practically no consideration for sequence homology. The error-prone NHEJ promotes accumulation of DNA damage, and its activity is a major driver for PARP1i synthetic lethality in HR-defective cells (31).

The different mechanisms of action of trabectedin and olaparib with respect to DDRR genes imply that finding predictive factors of response to their combined treatment cannot be assumed to be a simple summation. The objective of the present translational, exploratory study, was to look for a potential predictive gene expression signature of response to trabectedin and olaparib in sarcoma patients, taking advantage of tumor specimens derived from patients treated with this combination in the phase Ib TOMAS trial (13).

## Materials and methods

### Patient-derived samples

Patient samples were all derived from the Phase Ib TOMAS study patient cohort. Only patients treated at or above the third dose level were selected (trabectedin 0.920 mg/m^2^ q21d, olaparib 200 mg BID). The available material included pre-treatment biopsies or surgical specimens (formalin-fixed, paraffin-embedded; FFPE). All enrolled patients gave written, signed informed consent for the use of tumor samples for biomarker and exploratory analyses. The clinical study protocol was approved by the Institutional Review Board (IRB) and Ethics Committee of each participating center. All study procedures were performed in accordance to the Declaration of Helsinki.

### DNA and RNA extraction; DNA data analysis

DNA was extracted from patients’ specimens as previously described (13). DNA purity was checked by NanoDrop™ (Thermo Fisher Scientific, Life Technology, Monza, Italy). DNA concentration was determined by the Qubit dsDNA BR (broad range) and HS (high sensitivity) assay kits (Thermo Fisher Scientific) and the Qubit^®^ 3.0 Fluorometer (Thermo Fisher Scientific). DNA fragmentation was assessed by gel electrophoresis and by 2100 Bioanalyzer Instrument, with High Sensitivity DNA assay Kit (Agilent Technologies, Agilent Technologies, Inc., Santa Clara, California).

Good quality DNA samples underwent whole exome sequencing (WES) using the Twist Bioscience^®^ Human Core Exome (Consensus CDS) + IntegraGen content, for a genomic target of 37 Mb by IntegraGen SA (IntegraGen SA, Evry, France), and Novaseq 6000 sequencer (Illumina) with an average sequencing depth of 135X depth per exome and a coverage >98% at >50X. Genetic discovery analysis was performed by an in-house NGS pipeline (32, 33), constructed for WES analyses of paired cancer genomes in order to call somatic variations, indels and copy number alterations (CNA).

By means of publicly available databases (ClinVar) and PolyPhen-2 prediction tool (34), all benign variants were filtered out from the genetic analysis. Mutations were first looked into in the ClinVar database; if pathogenic, no further analysis was performed. If one mutation was found as being of unknown significance in the ClinVar database, or not described at all, PolyPhen-2 prediction tool was used to inquire its potential detrimental effect on protein function. Lower-quality DNA samples were analyzed with Oncomine-Comprehensive cancer panel v3 (Thermo Fisher Scientific) and meaningful alterations were filtered in with Ion ReporterTM 5.18.2.0 (Thermo Fisher Scientific).

RNA was extracted from FFPE samples with Maxwell^®^ RSC FFPE Kit (Promega Corporation, Madison, WI, USA) and Maxwell^®^ RSC Instrument. RNA purity, concentration and fragmentation were determined using DeNovix DS-11+ Spectrophotometer (DeNovix Inc., Wilmington, DE, USA), Qubit^®^3.0 Fluorometer (Invitrogen by Life Technologies, Eugene, Oregon, USA) and the Agilent 2100 Bioanalyzer System (Agilent Technologies, Wilmington, DE, USA), respectively.

### Nanostring^®^ nCounter assay

Expression of DDRR genes in tumor samples, was determined by NanoString^®^ nCounter Technology (NanoString Technologies, Seattle, WA, USA) by means of the nCounter^®^Vantage 3D™ RNA DNA Damage and Response Panel. Following manufacturer’s instructions, samples were prepared for hybridization, processed in the Prep Station, counted by the nCounter^®^ Analysis System.

### Quantitative real-time polymerase chain reaction and droplet digital absolute qPCR

For Real-time PCR analysis, 1 µg of total RNA was reverse-transcribed into cDNA using SuperScript IV VILO Master Mix (Thermo Fisher Scientific). TaqMan PCR analysis was performed with TaqMan Gene Expression Master Mix by means of ABI PRISM 7900HT System (Applied Biosystems, Monza, Italy). Taqman probes (Thermo Fisher Scientific) were as following: CDKN2A (Hs00923894_m1), APEX2 (Hs00205565_M1). Fluorescence data were automatically converted into Ct (cycle threshold) values. Data export (threshold 0.20) and analysis was performed by Microsoft Office Excel. Expression data were normalized to the geometric mean of housekeeping genes. For housekeeping genes, the Taqman probes (Thermo Fisher Scientific) were as following: B2M (Hs00984230_m1), UBC (Hs00824723_m1), GAPDH (Hs99999905_m1), ACTB (Hs99999903_m1).

The search for *CDKN2A* gene copy number was carried out by droplet digital PCR (ddPCR) as follows: DNA isolated from FFPE tumor tissues (as described above) was amplified using ddPCR Supermix for Probes (Bio-Rad, Hercules, CA, USA), using *CDKN2A* and housekeeping genes (*EIF2C1*, *AP3B1*, *RPP30*) probes (Bio-Rad, Segrate, Italy), according to manufacturer’s protocols as described in (35).

### *In situ* hybridization

The RNAscope^®^ Assay was used for *in situ* hybridization on FFPE tissue following standard protocol procedures (36). Specific preparation and pre-treatment included target retrieval lasting 10-15 minutes and Protease Plus incubation for 30 minutes.

### Immunohistochemistry

CDKN2A/p16 immunohistochemistry (IHC), on FFPE tumor tissues, was performed with a BOND-MAX automated staining platform (Leica Biosystems, Buccinasco, Italy), according to standard procedure. The specimens were sectioned at a thickness of 3 μm and stained on glass slides baked at 60°C for 30 minutes. Deparaffinization, rehydration and antigen retrieval were performed by Bond Dewax Solution, Bond Wash Solution, ethanol and Bond ER Solution 1 (prediluted; pH 6.0) antigen retrieval solution (Leica), performed on the BOND-MAX automated slide stainer (Leica) for 30 minutes at 95°C. The ready-to-use primary CDKN2A/p16 primary anti-human antibody (6H12; Leica), was incubated for 20 minutes at room temperature, followed by visualization with the Bond Polymer Refine Red Kit (Leica). The specimens were counter-stained with hematoxylin. Slide fixation was performed with mounting medium and observation under optical microscope (Leica DM750) equipped with Leica ICC50W camera (Leica).

### Statistical analyses

The nSolver software v3.0 was used to normalize the number of transcript copies with the geometric mean of 12 housekeeping genes. Log2-fold changes in gene expression were calculated comparing gene expression of samples from non-responder with responder patients. A patient was defined as responder in presence of a progression-free survival (PFS) ≥6 months. Differential expression analysis was performed using nSolver Advanced Analysis software (version 4.0, NanoString Technologies, Seattle, Washington, US), using the Differential Expression module (DE) in default settings. A gene was defined differentially expressed in a significant way, if associated with a p-value <0.05. Volcano plot was generated using the EnhancedVolcano R package v1.8. Volcano plot displays each gene’s -log10(p-value) and log2 fold change with the selected covariate. Highly statistically significant genes fall at the top of the plot above the horizontal lines, and highly differentially expressed genes fall to either side. Horizontal lines indicate various False Discovery Rate (FDR) thresholds or p-value thresholds if there is no adjustment to the p-values. Genes are colored if the resulting p-value is below the given FDR or p-value threshold.

Concerning the analysis of RNA ISH and IHC data, to compare expression levels between the two patient groups, an expert pathologist blinded to the treatment groups evaluated the staining intensity and the percentage of positive cells. Score 1: <25% positive cells, mild intensity staining; score 2: 25%< positive cells<50%, mild intensity staining; score 3: 50%<positive cells<75% strong intensity staining; score 4: positive cells >75%, strong intensity staining. Chi square Test was applied to calculate p value for ISH analysis: Wilcoxon rank-sum test was performed to compare the percentage of p16 positive IHC expression in responder *vs.* non-responder patients and calculate p-value.

### Bioinformatic analyses

We analyzed the genomic data that had been generated for the TOMAS study, to match the RNA expression data, looking for any genetic alteration that could affect DDRR gene function. We broadened our analysis to all known DDRR and related genes and looked both into point mutations and copy number variation alterations.

Analysis of genomic and transcriptomic data of 255 primary sarcoma samples from The Cancer Genome Atlas (TCGA) was performed using CBioPortal v3.6.17 (37), considering the dataset named “Sarcoma (TCGA, PanCancer Atlas)”. 249 soft tissue sarcoma samples were considered for survival analysis, excluding samples lacking complete genomic and expression data, and desmoid tumor samples. A gene was considered altered in a tumor sample if associated with a somatic mutation, a gene copy number alteration, or associated with an expression level higher/lower than two standard deviations (|z-score| > 2) with respect to the mean expression measured in diploid samples. CBioPortal was used also to visualize data of the TCGA sarcoma cohort, for retrieving patient clinical information, gene expression and CNV data. Survival analysis was performed with *survival* v3.2.13 and *survminer* v0.4.9 R packages.

## Results

### Patients’ demographics and analysis of DDRR gene mutations

The characteristics of patients eligible for the analyses are described in **Table 1**. 32 patients were included in the analysis. Bone sarcomas were a small fraction of the cohort, with only two cases of Ewing’s sarcoma (6%) and one osteoblastic osteosarcoma (3%). Concerning STS, the most prevalent histology was leiomyosarcoma (LMS; n=11), followed by synovial sarcoma (SS; n=5), liposarcomas (LPS; n=5), malignant peripheral nerve sheath tumors (MPNST; n=2) and undifferentiated pleomorphic sarcomas (UPS; n=2). The four remaining histotypes (grouped under the term “other”) included one malignant phyllodes tumor of the breast (MPT), one malignant myoepithelioma of the upper limb, one pleural solitary fibrous tumor and one myxofibrosarcoma of the limb.

**Table 1:** Patients’ demographics and tumor characteristics.

Gender	N (%)
Male	16 (50)
Female	16 (50)
**Age at protocol start**	
Median age, years (range)	61 (21-80)
**Histotype**	**N (%)**
Ewing’s sarcoma (ES)	2 (6)
Osteoblastic osteosarcoma (OS)	1 (3)
Leiomyosarcoma (LMS)	11 (34)
Synovial sarcoma (SS)	5 (16)
Liposarcoma (LPS)	5 (16)
*Dedifferentiated Liposarcoma (DDLPS)*	3
*Myxoid liposarcoma (MLPS)*	1
*Pleomorphic Liposarcoma (PLPS)*	1
Malignant peripheral nerve sheath tumor (MPNST)	2(6)
Undifferentiated pleomorphic sarcoma (UPS)	2 (6)
Other	4(13)
**Anatomic location of primary tumor**	**N (%)**
limb	18 (56)
uterus	7 (22)
retroperitoneum	4 (13)
pleural	1 (3)
breast	1 (3)
spine	1 (3)
**Grade**	**N (%)**
G2	3 (9)
G3	29 (91)
**Disease stage at protocol start**	**N (%)**
Locally advanced inoperable	3 (9)
Metastatic	29 (91)
*Metastases – anatomic location*	
Lung	29 (100)
Liver	9 (31)
Bone	12 (41)
Lymph nodes	3 (10)
Soft tissues	3 (10)

Among both responder and non-responder patients, we filtered out all benign or uncertain variants and found a few damaging, pathogenic mutations in DDRR genes. Considering the responder patient cohort (**Table 2**), patient 10 (TOMAS-10), affected by metastatic uterine LMS, had one *TP53* (pathogenic; ClinVar) and one (probably damaging; PolyPhen-2) *ERCC2* mutation. Other two *TP53* variants were detected in two LMS patients (one uterine and one retroperitoneal LMS); S215N being likely pathogenic/of unknown significance, and I195T being pathogenic, as described in ClinVar for both missense mutations. One “probably damaging” *ERCC6* mutation (as predicted by PolyPhen2 tool, being of unknown significance in the ClinVar database) was present in the tumor sample of one uterine LMS patient, and one MLPS patient’s tumor harbored both one pathogenic (ClinVar) *PTEN* mutation, and one *PIK3CA* mutation (possibly damaging, according to PolyPhen-2). One probably damaging (PolyPhen-2) *PIK3CA* mutation was observed also in TOMAS-39 patient, affected by malignant phyllodes tumor (MPT).

**Table 2:** Likely pathogenic mutations in DDRR and related genes among responder patients.

**Responders**	***TP53***	***ERCC2***	***ERCC6***
	G245S (TOMAS-10; LMS_UT)G245S (TOMAS-10; LMS_UT)I195T (TOMAS-44; LMS_RP)	G615W (TOMAS-10; LMS_UT)	E272K (TOMAS-41; LMS_UT)
	***PIK3CA*** R93W (TOMAS-38; MLPS_LIMB)P104S (TOMAS-39; MPT)	***PTEN*** R173C (TOMAS-38;MLPS_LIMB)	

UT, uterus; RP, retroperitoneum.

Among non-responder patients (**Table 3**), *TP53* mutations were found in two non-responder patients affected by metastatic uterine LMS (C242S and Y205D), both of uncertain pathogenicity according to ClinVar, but probably damaging according to PolyPhen-2. One mutation predicted as “damaging” on BRCA1 protein function (by Polyphen2; of uncertain significance according to ClinVar) was detected in patient TOMAS-29 (metastatic synovial sarcoma of the lower limb). Another patient affected by metastatic synovial sarcoma had a *RAD51C* missense mutation (pathogenic/likely pathogenic in ClinVar). Patient TOMAS-25 had three deleterious *ARID1A* indels, while patient TOMAS-26 tumor sample harbored a gain-of-function mutation in the *ERBB2* gene, predicted as “possibly damaging” on protein function.

**Table 3:** Likely pathogenic mutations in DDRR and related genes in non-responder patients.

**Responders**	***TP53***	***ERCC2***	***RAD51C***
	C242S (TOMAS-32; LMS_UT)Y205D (TOMAS-34; LMS_UT)	T231M (TOMAS-29; SS_LIMB)	L138F (TOMAS-21;SS_LIMB)
	***ERBB2*** R678W(TOMAS-26; MPNST_LIMB)	***ARID1A*** Three deleterious indels(TOMAS-25; UPS_LIMB)	

UT, uterus; RP, retroperitoneum.

Gene copy number analysis was performed to identify differences among the two groups (**Table 4**). Dedifferentiated liposarcomas showed *MDM2/CDK4* gene amplifications, as already detected by diagnostic cytogenetics (TOMAS-43, TOMAS-48; both responder patients). Significant differences in copy number gain in *CDKN2A* gene (evaluated in comparison to housekeeping genes) was reported for responder in comparison to non-responder patients (paired Student’s t-test; p=0.038). *MYC* gene amplification was detected in one responder patient affected by malignant myoepithelioma, and *MYC* amplification associated with *HEY1* amplification (possibly due to close chromosomal location) was identified in patient TOMAS-34 (non-responder patient affected by uterine LMS). Finally, *ERBB2* amplification was observed in one case of non-responder UPS of the limb.

**Table 4:** Copy number differences in DDRR and related genes among responder and non-responder patients.

**Responders**	***CDKN2A***	***MDM2***	***CDK4***	***MYC***
	2.45 (TOMAS-10; LMS_UT)1.44 (TOMAS-18; SS_LIMB)1.9 (TOMAS-30; LMS_UT)2.64 (TOMAS-33; SS_LIMB)2.4 (TOMAS-48; DDLPS_RP)	38 (TOMAS-43; DDLPS_RP)20 (TOMAS-48; DDLPS_RP)	24 (TOMAS-43; DDLPS_RP)23 (TOMAS-48; DDLPS_RP)	5.08 (TOMAS-45; malignant myoepithelioma)
**Non-responders**	***CDKN2A***	***ERBB2***	***HEY1***	***MYC***
	1.3 (TOMAS -9; MPNST_LIMB)1.84 (TOMAS-17; SS_LIMB)1.42 (TOMAS-21; SS_LIMB)1.34 (TOMAS-34; LMS_UT)	5.56 (TOMAS-35; UPS_LIM	5 (TOMAS-34; LMS_UT)	5 (TOMAS-34; LMS_UT)

UT, uterus; RP, retroperitoneum.

### Differential expression of DDRR genes among responder and non-responder patients

**Figure 1:**
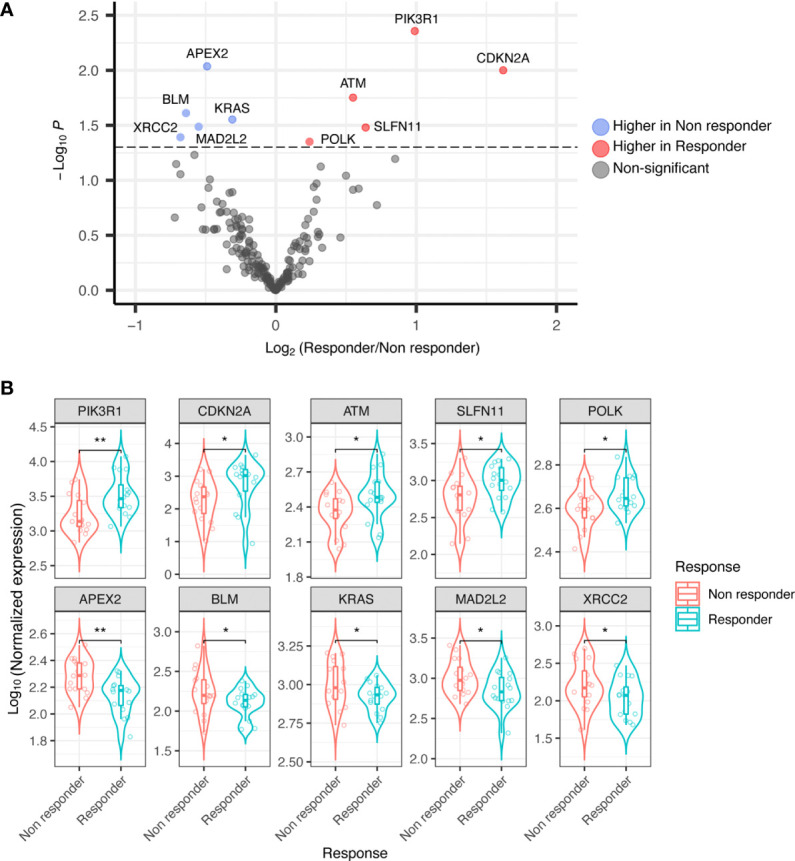
**(A)** Volcano plot showing differential expression of DDRR genes in responder *vs.* non-responder patients. **(B)** Boxplot showing differential expression of DDRR genes in responder *vs.* non-responder patients, with normalized expression. P-value by Wilcoxon Rank-Sum test. *p<0.05; **p<0.01.

Thirty-two RNA samples were extracted from FFPE archival tumor tissue from patients subsequently treated with trabectedin and olaparib combination. Significant differential expression levels of DDRR genes were found between the group of 16 responders (PFS ≥ 6 months), and that of 16 non-responders (PFS < 6 months). In detail, the expression of *CDKN2A, PIK3R1, SLFN11, ATM*, (and *POLK*) were significantly higher in responders; whilst *APEX2, BLM, XRCC2, MAD2L2*, and *KRAS* were significantly higher in non-responders (**Figures 1A, B**).

### Validation of biomarker expression by RNA-ISH, qRT-PCR and IHC

**Figure 2:**
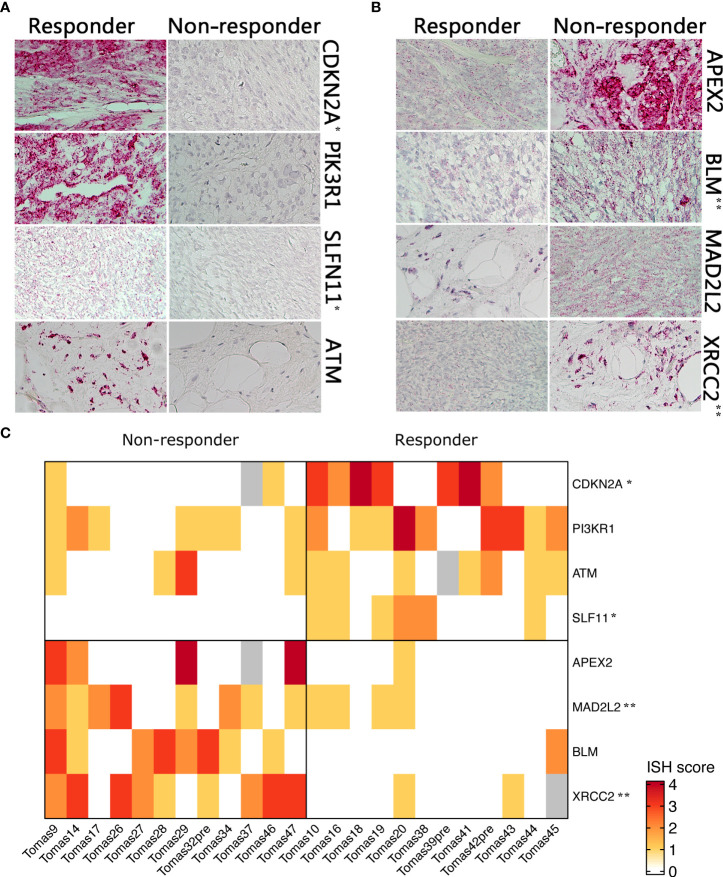
RNA ISH of selected genes in responder *vs.* non-responder patients. **(A)** Higher expressed genes in responder patients **(B)** higher expressed genes in non-responders **(C)**. Heatmap showing differential RNA ISH staining between responder and non-responder patients. ISH score was assigned by an expert pathologist on the basis of staining intensity and percentage of positive cells. P-value was calculated by Chi-square test. *p<0.05; **p<0.01.

Differential expression of selected candidate biomarkers and their exact subcellular and tissue localizations were analyzed by RNA-ISH. Specific probes for *CDKN2A*, *PIK3R1, SLFN11*, and *ATM* were more hybridized in tissue slices from tumors of responder patients than in those samples derived from non-responder patients (**Figure 2A**). *APEX2, BLM*, *XRCC2*, and *MAD2L2* were less hybridized in tissue slices from responder patient-derived tumors than in those ones from non-responder patients (**Figure 2B**). A heatmap was generated based on the ISH scores, displaying the differential expression of the eight identified genes among the two patient groups (**Figure 2C**).

**Figure 3:**
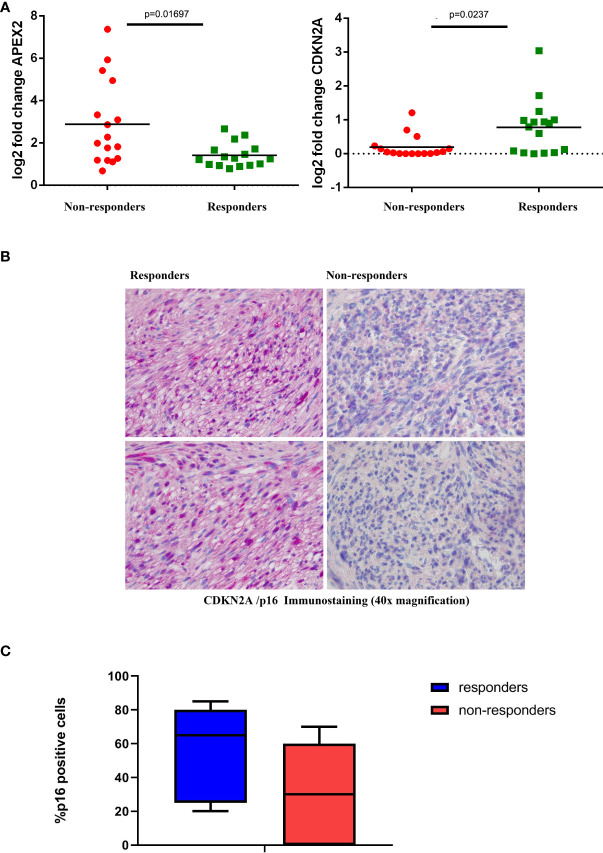
Validation Assays **(A)** Expression levels of representative genes (CDKN2A, left; and APEX2, right) among responder and non-responder patients. Statistically significant differential expression was shown between the two groups (Wilcoxon rank-sum test). **(B)** Representative IHC staining of CDKN2A/p16 in tumor samples from responder and non-responder patients. **(C)** Box plot distribution of CDKN2a/p16 expression level (percentage of IHC positive cells) in responders and non-responders patients.

The expression levels were further confirmed by qRT-PCR (**Figure 3A**) and also at the protein level in terms of IHC expression for CDKN2A/p16, where a significant difference was detected between responders and non-responders (p=0.041; **Figures 3B, C**).

### Correlation of candidate biomarker gene expression levels and overall survival in TCGA sarcoma cohort

**Figure 4:**
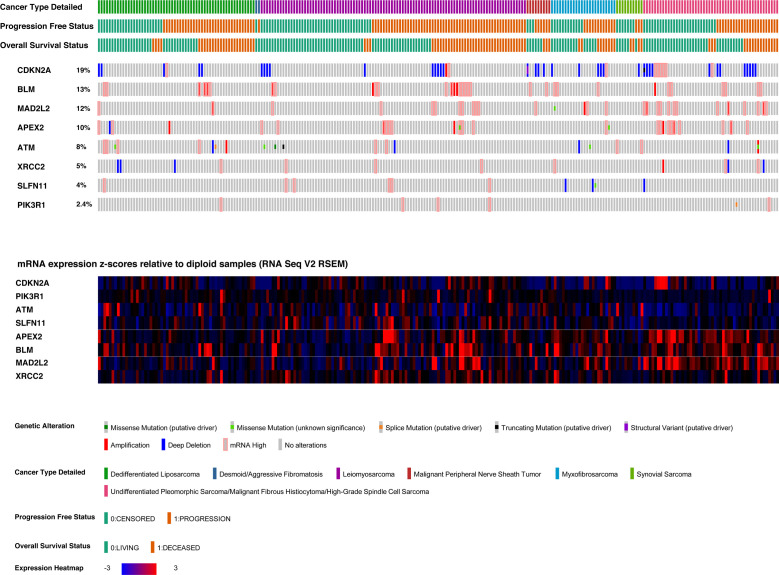
Oncoprint and heatmap of candidate biomarkers in the TCGA sarcoma cohort.

The sarcoma dataset was derived from genomic and expression analysis of 255 sarcoma samples from the TCGA sarcoma cohort. 249 samples were selected, being the ones with all data of interest available, and excluding desmoid tumors from the dataset, given their peculiar clinical-pathological behavior (37). The gene characterized by the highest number of genomic or transcriptomic alterations (**Figure 4**) was *CDKN2A* (altered in 19% of patients), followed by *BLM* (altered in 13% of patients), and *MAD2L2* (altered in 12% of patients). The most frequent *CDKN2A* alteration was homo-deletion (n=38, 15% of patients).

**Figure 5:**
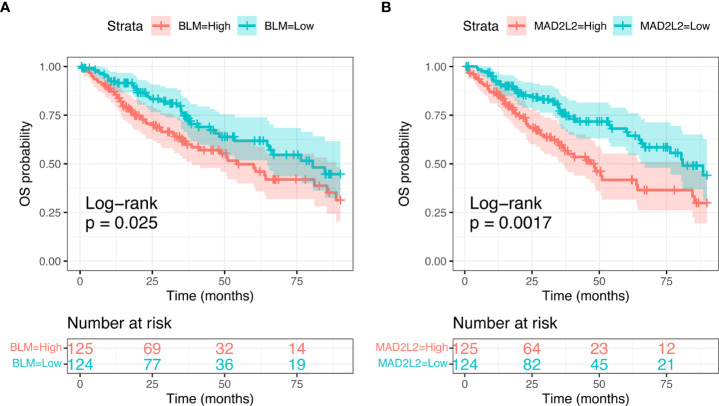
Kaplan-Meier curves showing Overall Sirvival in the sarcoma TCGA cohort, according to selected candidate biomarker genes (BLM, **A**, and MAD2L2, **B**).

We subsequently focused on differences in expression levels of the eight identified candidate genes in the sarcoma cohort of TCGA dataset, to look for any correlation with survival outcomes. We found a significant relation between *MAD2L2* (Log-rank; p=0.0017) and *BLM* (Log-rank; p=0.025) expression levels and OS (**Figure 5**). The expression levels of the other six genes were not significantly related to OS (**Supplementary Figure 1**).

## Discussion

Our work has focused on a relevant translational research question stemming from the clinical results of the phase Ib TOMAS trial (13), asking whether there might be any way to predict response to trabectedin+olaparib treatment in BSTS patients. Of course, the answer to this question is a multi-factorial, poly-genic one, especially considering the low prevalence of BRCA1/2 defects in BSTS (38). Indeed, we identified few differentially expressed DDRR genes, which could provide the basis for a “personalized-medicine” approach to sarcoma treatment with this combination.

We analyzed WES and targeted-panel NGS data of patients from the TOMAS study, for whom RNA expression data were also available (32 patients), looking for any mutation, indel or CNV in DDRR genes, to integrate the expression signature with any mutational input which would not modify expression levels, but could alter gene function as well. Indeed, loss of function gene mutations might have the same effect of reduced gene expression for a given gene, while gain of function could correspond to gene overexpression. Indeed, we observed some relevant DDRR genes mutations, indels and CNVs in both responder and non-responder patients.

We then moved to expression profile analysis and identified a difference in terms of DDRR gene expression between 16 responder and 16 non-responder patients from the phase Ib TOMAS trial. We found an 8-gene signature of differentially expressed DDRR genes which significantly correlates with better outcome in patients treated with trabectedin+olaparib combination, separating our patient population in two groups according to PFS (longer or shorter than 6 months). The differential gene expression data were corroborated with ISH data, confirming expression at the sub-cellular level with RNA *in situ* hybridization technique, and also at the protein level (*e.g.* CDKN2A/p16). Our signature emerged from a broad DDRR panel, including 180 genes.

*CDKN2A, PIK3R1, SLFN11, ATM* were characterized by significantly higher expression levels in responder patients; *APEX2, BLM, XRCC2, MAD2L2* displayed instead significantly higher expression levels in non-responder patients. Each gene deserves a separate discussion, being implicated in different aspects of DNA damage response and repair cellular machinery.

*CDKN2A* is a well-known tumor suppressor gene with a pivotal role in cell cycle control, slowing down G1 to S phase progression. It is involved in DDRR, and its low expression is also a negative prognostic factor across several tumor types (39–43). Similarly, low expression levels of *PIK3R1*, the gene encoding the regulatory subunit of *PIK3CA* (p85α), have been associated to poor prognosis, in particular in breast cancer (44). In the TCGA sarcoma cohort dataset, our analyses did not show any significant relationship between *CDKN2A* and *PIK3R1* expression and survival, suggesting that these two genes are unlikely prognostic factors in STS and potentially might be involved in response to the treatment in this case series.

Considering *SLFN11, ATM, APEX2, BLM, XRCC2* and *MAD2L2* expression, a functional role in response to trabectedin+olaparib treatment might be hypothesized based on their biological roles. *SLFN11* and *ATM* showed higher expression levels in responder patients. *SLFN11* had already been associated to PARPi response (45). SLFN11 enhances cancer cell sensitivity to DNA-damaging agents (46), through a peculiar mechanism. Indeed, *SLFN11* prevents the synthesis of proteins, which are crucial for cell survival upon significant extents of DNA damage. Namely, *SLFN11* downregulates type II RNAs, inducing reduced translation of DDRR genes such as *ATM* and *ATR* (47). In this view, *ATM* higher expression in responder patients seems controversial, because it would lead to a more HR-proficient tumor cell profile in terms of DDRR response. *APEX2* is a base-excision repair apurinic/apyrimidinic endonuclease (48). In multiple myeloma cells, it has been described as a key regulator of HR (49). Hence, lower *APEX2* expression in responder patients is consistent with HR impairment and better response to trabectedin+olaparib response. What is more, *APEX2* has been described as synthetic lethal in cells bearing BRCA2 defects (50). Considering *BLM*, its role in HR is well-known, both for initiation of HR upon DSBs and for Holliday junction dissolution at the end of the repair process (51). Hence, its lower expression in responder patients could have a direct implication driving sarcoma cells towards a HR-deficient phenotype. *XRCC2* is also involved in DSBs repair by HR (52). In our patient population, expression was consistently higher in non-responder patients compared to responders. Given the relevance of HR deficiency for both olaparib and trabectedin mechanism of action, *XRCC2* role in resistance to trabectedin+olaparib treatment could be at least partially explained. *MAD2L2*, instead, has a more prominent role in NHEJ (53). As anticipated, NHEJ does not affect trabectedin efficacy in a relevant way. Theoretically, proficient NHEJ might influence PARPi action, supporting our observation that responder patients show lower levels of *MAD2L2* expression. Both *XRCC2* and *MAD2L2* higher expression was associated with worse survival in the TCGA sarcoma cohort.

Looking for potential DDRR gene function differences, which might not be reflected in expression levels, a few noteworthy mutations have emerged from analysis of WES and targeted-panel NGS data from the TOMAS phase Ib study. Indeed, apart from the expected *TP53* mutations, which we detected in our LMS patients, we observed one *ERCC2* mutation in one responder patient (**Tables 2**, **3**), who displayed a mutation resulting in a G615W amino acid substitution, predicted as “probably damaging” (PolyPhen2 score of 1). *ERCC2* is involved in TC-NER, so a loss of function mutation could represent a “resistance mechanism” to trabectedin. This mutation might have represented our patient’s Achilles’ heel to maintain a sustained response (PFS=10 months). One responder affected by a metastatic myxoid liposarcoma of the lower limb carried a mutation resulting in the R173C *PTEN* amino acid substitution (TOMAS-38), which is a loss of function mutation. Indeed, *PTEN* mutations have been described as synthetic lethal with PARPi (54). Among DDRR genes, we also included *ERBB2* for its potential effects in DNA damage and repair pathways. We found *ERBB2* gene amplification in one patient affected by metastatic UPS of the lower limb, and one gain of function point mutation resulting in the amino acid substitution R678W in a patient affected by metastatic MPNST of the lower limb. *ERBB2* amplification has already been reported in UPS (55), as well as *ERBB2* gain of function in MPNST (56). The specific R678W substitution, falling into *ERBB2* transmembrane domain, confers significant cell survival advantage with respect to wild-type *ERBB2* (57). Intriguingly, it has been found that *ERBB2* expression affects the repair of specific DNA lesions produced by chemotherapy, linking *ERBB2* to the DNA damage and repair response (58).

In conclusion, the response of BSTS patients to trabectedin and olaparib combination correlates with the expression of DDRR genes. *CDKN2A, PIK3R1, SLFN11, ATM* and *APEX2, BLM, XRCC2, MAD2L2* differential expression discriminates responder and non-responder patients. The predictive role of these potential biomarkers warrants further investigation; we will explore this gene signature within data derived from our ongoing TOMAS-2 multicentric, randomized, phase II study.

## Data availability statement

The original contributions presented in the study are included in the article/**Supplementary Material**. Further inquiries can be directed to the corresponding authors.

## Ethics statement

The studies involving human participants were reviewed and approved by Institutional review board - Candiolo Cancer Institute, str. prov 142 km 3.95 Candiolo, Italy. The patients/participants provided their written informed consent to participate in this study.

## Author contributions

Conceptualization: GGr, YP; Methodology: MLC, GF, UM, EB, YP, GCr; Formal Analysis: MLC, UM, EB, GF, YP, CI, CL, AP, IS; Investigation: AM, MLC, AP, GCh, EM, GGi, SB, LDA, MS, APDT; Visualization: CL, UM, EB, GF, YP; Writing - Original Draft Preparation, AM, YP; Writing - Review & Editing, AM, DS, GGr, GF, YP, MA, DS; Supervision, YP, GGr; Project Administration, YP, GGr; Funding Acquisition, GGr, DS, YP, AB, LDA. All authors have read and agreed to the published version of the manuscript.

## Funding

This work was supported by RC 2019 Ministero della Salute; AIRC IG 23104 to GGr, Alleanza contro il cancro-working group Sarcomi, Ricerca Corrente-Reti 2021 RCR 2021 WP8 to YP; FPRC 5x1000 Ministero della Salute 2015 ImGen to GGr and to DS, FPRC 5xmille MIUR 2014 to GGr.; Fondazione per la ricerca sui tumori dell'apparato muscoloscheletrico e rari ONLUS CRT RF = 2016 -0917; AIRC IG 20259 to DS; Ministero della Salute-Ricerca Finalizzata- Giovani Ricercatori GR-2016-02362726 to YP; AIRC 5 per Mille 2018 - ID. 21091 to AB; AIRC IG 2018 - ID.21923 to AB; MIUR BiLiGeCT - Progetto PON ARS01_00492 to AB; EB was the recipient of a PhD fellowship from Department of Medical Sciences, University of Torino (“Dipartimenti di Eccellenza 2018-2022”, Project no. D15D18000410001), F.C. was supported by AIRC fellowship “Volontari Comitato Abruzzo-Molise” Rif. 21173.

## Acknowledgments

TOMAS 1b clinical trial is an Italian Sarcoma Group study. The results shown here are in part based upon data generated by the TCGA research network: https://www.cancer.gov/tcga.

## Conflict of interest

GGr has received fees for consulting/advisory roles from PharmaMar, Lilly, Novartis, Bayer, and Eisai. LDA received travel grant from PharmaMar and Lilly. MA has received fees for consulting/advisory roles from Bristol- Myers Squibb, Merck, and Roche. AB served in a consulting/advisory role for Illumina and Inivata. AB is cofounder and shareholder of NeoPhore. AB is a member of the NeoPhore scientific advisory board.

The remaining authors declare that the research was conducted in the absence of any commercial or financial relationships that could be construed as a potential conflict of interest.

## Publisher’s note

All claims expressed in this article are solely those of the authors and do not necessarily represent those of their affiliated organizations, or those of the publisher, the editors and the reviewers. Any product that may be evaluated in this article, or claim that may be made by its manufacturer, is not guaranteed or endorsed by the publisher.
