# Supplementary figures and images for: DNA damage response and repair genes in advanced bone and soft tissue sarcomas: An 8-gene signature as a candidate predictive biomarker of response to trabectedin and olaparib combination
Source: Front Oncol. 2022 Aug 30;12:844250. doi: 10.3389/fonc.2022.844250 (PMC9469659; doi:10.3389/fonc.2022.844250)

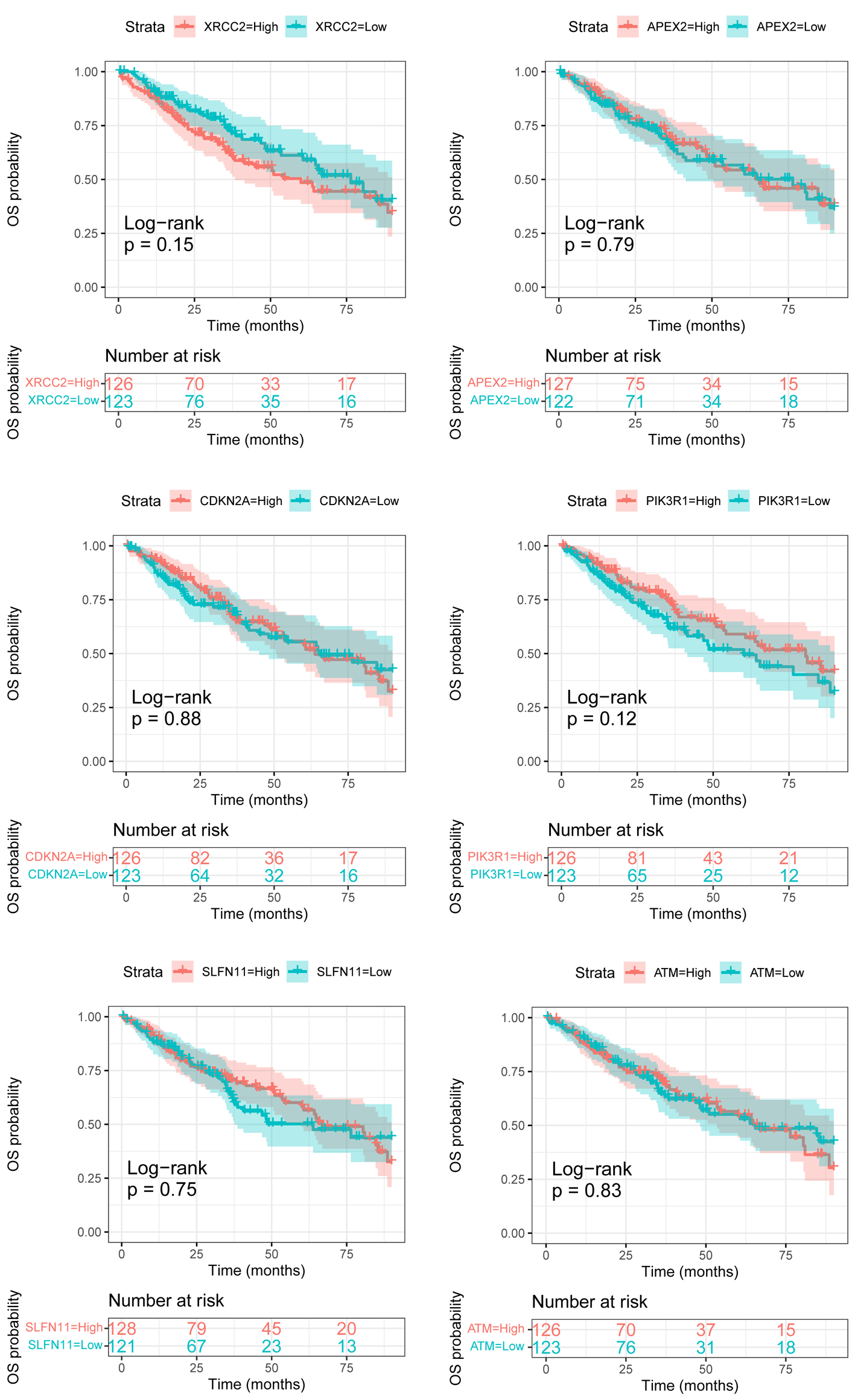

Supplement: Supplementary file 1 [file Image_1.tif]
